# Nutrition: A Primary Therapy in Pediatric Acute Respiratory Distress Syndrome

**DOI:** 10.3389/fped.2016.00108

**Published:** 2016-10-13

**Authors:** Bryan Wilson, Katri Typpo

**Affiliations:** ^1^Department of Emergency Medicine, University of Arizona College of Medicine, Tucson, AZ, USA; ^2^Department of Pediatrics, Steele Children’s Research Center, University of Arizona College of Medicine, Tucson, AZ, USA

**Keywords:** pediatric, ARDS, nutrition, intensive care

## Abstract

Appropriate nutrition is an essential component of intensive care management of children with acute respiratory distress syndrome (ARDS) and is linked to patient outcomes. One out of every two children in the pediatric intensive care unit (PICU) will develop malnutrition or have worsening of baseline malnutrition and present with specific micronutrient deficiencies. Early and adequate enteral nutrition (EN) is associated with improved 60-day survival after pediatric critical illness, and, yet, despite early EN guidelines, critically ill children receive on average only 55% of goal calories by PICU day 10. Inadequate delivery of EN is due to perceived feeding intolerance, reluctance to enterally feed children with hemodynamic instability, and fluid restriction. Underlying each of these factors is large practice variation between providers and across institutions for initiation, advancement, and maintenance of EN. Strategies to improve early initiation and advancement and to maintain delivery of EN are needed to improve morbidity and mortality from pediatric ARDS. Both, over and underfeeding, prolong duration of mechanical ventilation in children and worsen other organ function such that precise calorie goals are needed. The gut is thought to act as a “motor” of organ dysfunction, and emerging data regarding the role of intestinal barrier functions and the intestinal microbiome on organ dysfunction and outcomes of critical illness present exciting opportunities to improve patient outcomes. Nutrition should be considered a primary rather than supportive therapy for pediatric ARDS. Precise nutritional therapies, which are titrated and targeted to preservation of intestinal barrier function, prevention of intestinal dysbiosis, preservation of lean body mass, and blunting of the systemic inflammatory response, offer great potential for improving outcomes of pediatric ARDS. In this review, we examine the current evidence regarding dose, route, and timing of nutrition, current recommendations for provision of nutrition to children with ARDS, and the current literature for immune-modulating diets for pediatric ARDS. We will examine emerging data regarding the role of the intestinal microbiome in modulating the response to critical illness.

## Introduction

There are direct nutritive and non-nutritive benefits of feeding patients with pediatric acute respiratory distress syndrome (pARDS). Adequate nutrition therapy in support of protein, energy, and micronutrient needs prevents loss of lean body mass, improves protein turnover for production of acute phase and immune proteins, prevents depletion of tissue antioxidant systems which occur with starvation, and is associated with improved 60-day mortality in mechanically ventilated, critically ill children (1–3). Adequate protein delivery prevents loss of respiratory and cardiac muscle function and is associated with increased ventilator-free days and improved mortality in pARDS (4). And, yet, median delivery of enteral nutrition (EN) remains 40–75% of goal over the first week of pediatric intensive care unit (PICU) hospitalization (3, 5–10). In addition, as protein synthesis rates are reliant upon adequate protein delivery, patients may not realize benefits of adjuvant therapies that rely on protein signaling if they fail to meet goal EN.

The gastrointestinal tract is a primary lymphoid organ, housing 70% of all immune cells with the ability to alter systemic inflammatory responses (11–14). Non-nutritive benefits of feeding, therefore, include downstream reduction in pro-inflammatory signaling to the lung (11, 15, 16). The gastrointestinal tract is an important target to improve lung inflammation, during pARDS, and subsequent patient outcomes. The intestinal epithelial barrier is a single-cell monolayer, which must absorb fluids and nutrients, interact with commensal organisms, and prevent entrance of pathogens and their toxic products (17). Intestinal barrier dysfunction has many downstream negative consequences. It is associated with bacterial translocation, endotoxemia, organ failure, immune dysfunction, and lung inflammation (13, 18, 19). The intestinal epithelial barrier, the host microbiome, and intestinal immune system interaction contribute to the pathophysiology of pARDS and present novel therapeutic targets.

Specific nutrients provided at pharmacological doses or nutrition supplemented with immune-modulating factors may directly impact lung pro-inflammatory cytokines and neutrophil accumulation in the setting of pARDS (20–22). Non-nutritive goals of feeding with either a standard enteral formula or an immune-modulating formula include maintenance of intestinal barrier functions, to alter or attenuate the immune or inflammatory responses in pARDS, and to modulate microbiome and/or host–microbe interactions. The host microbiome is emerging as an important mediator in chronic as well as acute inflammatory states. An understanding of the necessary and sufficient nutrition for each of these nutritive and non-nutritive benefits is important to determine the minimum necessary macronutrient and micronutrient delivery during each phase of pARDS to optimize patient outcomes. Optimizing nutritional therapy to delivery the right nutrition to the right patient at the right time is an excellent opportunity to improve outcomes in pARDS.

## Metabolism During Pediatric Critical Illness

**Figure 1:**
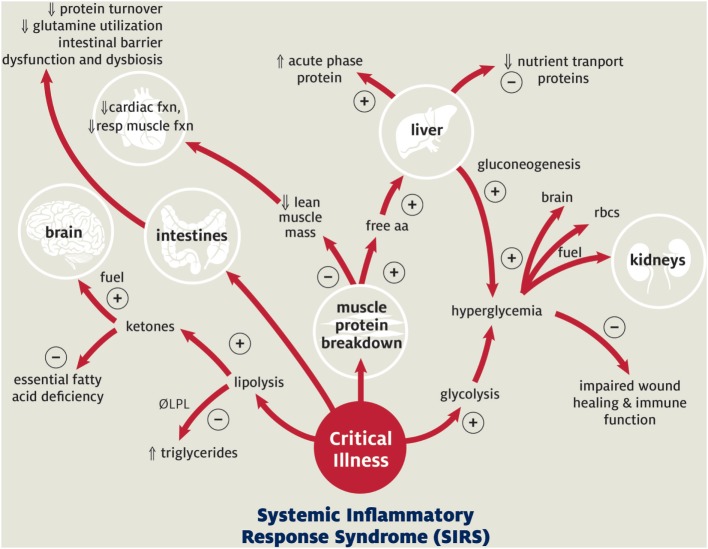
**Metabolic response to pediatric critical illness**. + Acute adaptive response; − maladaptive consequence; 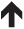 increased; 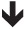 decreased. Abbreviations: LPL, lipoprotein lipase; aa, amino acids; resp, respiratory; rbc’s, red blood cells. Adapted with permission from Graciano and Turner (25). Copyright from the Society of Critical Care Medicine.

Currently, knowledge of the normal nutritional needs of children and of the metabolic response to critical illness guides recommendations and goals for nutritional support in pediatric patients (Figure 1). Critical illness and the associated inflammation and tissue injury alter metabolism by inducing a catabolic state, which may exacerbate pre-existing malnutrition (1, 23). Metabolism shifts away from growth to support the production of acute phase proteins, enzymes, and glucose to facilitate recovery (23, 24). This is accomplished through glycolysis, lipolysis, and protein turnover with resulting hyperglycemia, ketone production, and breakdown of skeletal muscle protein (23, 24). This diversion of resources results in cessation of normal growth and development in pediatric patients (23, 24). Prolonged persistence of this catabolic state progressively depletes the body’s nutritional resources, ultimately leading to muscle wasting, impaired cardiopulmonary function, decreased immune response, and poor wound healing (Figure 1). Pediatric patients (especially those with pre-existing nutritional, cardiopulmonary, or muscular disease) are particularly susceptible to these adverse sequelae due to their limited macronutrient and micronutrient reserves. In the setting of pARDS, this loss of lean body mass and diaphragmatic function is likely to amplify the severity of the patient’s respiratory failure and prolong ventilator dependence.

## Pre-Existing Nutritional Status Mediates Patient Outcomes

Patients who are undernourished prior to their critical illness experience worse outcomes (Table 1) (26). High quality research to understand how adequacy of nutrition delivery and premorbid nutritional status influences outcomes of pARDS is limited; however, pre-illness nutritional status and adequacy of macronutrient delivery early in critical illness are well established as risk factors for morbidity and mortality of critically ill patients in neonatal and pediatric populations (2, 3, 7, 10, 26–31). Malnutrition is both prevalent and incident in children hospitalized in the PICU; 30% of children admitted to the PICU have pre-existing acute or chronic malnutrition, and up to 58% of patients are discharged from the PICU malnourished (3, 6, 28). Even children who are well nourished prior to their critical illness may develop acute protein–energy malnutrition within 48 h of PICU admission due to limited protein and energy reserves in the setting of severe acute illness (6, 32–35). In a study of 1622 mechanically ventilated children, underweight nutritional status based on admission BMI *z*-score was associated with an increase in hospital-acquired infections, fewer ventilator-free days, and lower likelihood of hospital discharge (Hazard ratio 0.71, *p* < 0.001) (Table 1) (26). These high-risk patients may benefit from personalized nutritional strategies.

**Table 1:** **Key observational studies of the impact of nutrition and nutritional status on patient outcomes in pediatric critical illness**.

Study	Patient population	Exposure of interest	Outcomes
**Timing, dose, route nutrition**			
Wong et al. (4)	Multicenter study of 107 children with ARDS	Enteral calorie and protein adequacy	Reduction in ICU mortality in patients who received adequate calories (34.6 versus 60.5%, *p* = 0.025) and adequate protein (14.3 versus 60.2%, *P* = 0.002) compared with those who did not. Patients with adequate protein intake also had more VFDs [median (interquartile range), 12 (3.0–19.0) versus 0 (0.0–14.8) days; *p* = 0.005]
Mikhailov et al. (36)	Multicenter study of 5105 children 1 month to 18 years with PICU length of stay ≥96 h	Early enteral nutrition	Early EN was associated with lower odds of mortality (OR 0.52; 95% CI 0.34–0.76, *p* = 0.001). No significant differences in length of stay or duration of mechanical ventilation in patients exposed to early EN
Mehta et al. (2)	Multicenter study of 1245 children 1 month to 18 years requiring mechanical ventilation ≥48 h	Enteral protein adequacy	Adequacy of enteral protein intake was associated with lower 60-day mortality (*p* < 0.001)
Mehta et al. (3)	Multicenter study of 500 children 1 month to 18 years requiring mechanical ventilation >48 h	Enteral calorie adequacy	A higher percentage of goal energy intake *via* enteral nutrition route was significantly associated with lower 60-day mortality (*p* = 0.002). Mortality was higher in patients who received PN (*p* = 0.008). Patients admitted to units that utilized a feeding protocol had a lower prevalence of acquired infections and this association was independent of the amount of energy or protein intake
**Nutritional status**			
Bechard et al. (26)	Multicenter study of 1622 mechanically ventilated children from 2 study cohorts	Baseline nutritional status	BMI *z*-score classification as underweight or obese was associated with higher risk of hospital-acquired infections and lower likelihood of hospital discharge. Underweight children had a higher risk of mortality and fewer ventilator-free days
de Souza Menezes et al. (27)	Single-center prospective study of 385 critically ill children	Baseline nutritional status	Malnutrition (BMI *z*-score <−2 for weight for age, height for age, and BMI) was associated with greater length of mechanical ventilation and length of stay. Malnutrition was not a predictor of mortality
**EN safety and complications**			
Lopez-Herce et al. (37)	Single-center prospective, observational study of 526 critically ill children who received post-pyloric enteral nutrition (PEN)	Enteral nutrition	The stepwise multivariate logistic regression analysis showed that the most important factors associated with gastrointestinal complications were shock, epinephrine at a rate higher than 0.3 μg/kg/min and hypophosphatemia
Panchal et al. (38)	Multicenter study of 339 children admitted to PICU for ≥96 h and on vasoactive infusions	Enteral nutrition	Unadjusted mortality was lower in the patients exposed to EN (6.9 versus 15.9%, *p* < 0.01). Vasoactive-inotropic score did not differ between the two groups except on day 1. No difference in gastrointestinal outcomes between patients who did and did not receive enteral nutrition

*VFD, ventilator-free days; BMI, body mass index*.

A growing percentage of children worldwide are now obese (39). While literature on the obese and critically ill pediatric patient is sparse, adult studies demonstrate a complex relationship between obesity and clinical outcomes in critical illness and acute respiratory distress syndrome (ARDS) (40, 41). While overall morbidity, including ventilator days and length of hospitalization, is higher, mortality was lower (40, 41). A mortality benefit may be due to a higher nutritional reserve in the setting of prolonged catabolism (40, 41). Similar to risks in underweight children, obesity based on admission BMI *z*-score is associated with higher risk of hospital-acquired infections and lower likelihood of hospital discharge (hazard ratio 0.82, *p* = 0.04) (26). It is important to note, however, that obesity is not exclusive of malnutrition and that the obese child should receive the same consideration for nutritional supplementation (40). Both obese and underweight children are at increased risk compared with normal weight children for adverse outcomes while undergoing mechanical ventilation (26). For this reason, a nutritional support strategy should include early screening and diagnosis of malnutrition (42).

## Macronutrient Requirements During Pediatric Critical Illness

### Protein

Protein requirements during acute illness are increased secondary to the catabolic state generated to support the production of acute phase proteins, repair of tissue, and the production of immune proteins (1). Protein demands can be increased by as much as 100% in severe sepsis, and even mild stressors, such as routine surgery, may increase protein needs by 25% (1). Infants who require extracorporeal membrane oxygenation have the highest reported rates of protein turnover in the literature (43, 44). As discussed previously, prolongation of this catabolic state can result in decreased cardiopulmonary function secondary to breakdown of cardiac and respiratory muscle mass. While evidence currently suggests this catabolic process is not averted through protein intake but prevents recruitment of lean muscle mass as the major source of amino acids and increases protein production (44), and muscle mass can be preserved (45). Many adjuvant therapies, such as insulin for treatment of stress hyperglycemia, may be dependent upon adequate protein delivery to demonstrate efficacy. In 12 neonates on extracorporeal membrane oxygenation, Agus et al. found that improved protein balance as a result of insulin therapy was only seen in patients with adequate protein delivery (44). Liebau et al. was able to improve protein balance, but not protein breakdown, in 10 critically ill adults with sufficient enteral protein delivery (46). Concurrent supplementation with adequate carbohydrate and lipid energy sources improves protein turnover and synthesis, thereby potentiating this counterbalancing (47). The decrease in protein breakdown seen with glucose supplementation in simple starvation does not decrease muscle catabolism for gluconeogenesis in the response to critical illness (47). Excessive protein administration should also be avoided as it can result in morbidity, such as azotemia, metabolic acidosis, and neurologic dysfunction, particularly as the patient improves and catabolic turnover subsides (48, 49). Recommended protein requirements in critically ill children vary by age and range from 3 g/kg/day in infants to 1.5 g/kg/day in adolescents (50).

### Energy

Energy sources in the form of lipids and carbohydrates are needed to facilitate protein synthesis and meet the patient’s total energy expenditure, which includes the energy needed for basal metabolism, growth, physical activity, and thermogenesis. Critical illness induces an unpredictable metabolic state in children, which makes calculation of energy requirements challenging (51–53). Decisions regarding energy prescription are further complicated by the inherent variability both between patients and within the same patient over the course of their ICU stay, depending on ICU course and severity of illness (54–57). Age, anthropometric criteria, biochemical criteria, clinical exam, disease category, and stage of illness have all been demonstrated to be poor predictors of energy expenditure (51, 54). Other common factors that affect total energy expenditure and further complicate estimation are fever, sedation, temperature support, paralytics, and ventilator support. The variation in these factors requires frequent reassessment and adjustment of nutritional support to accurately meet but not exceed the patient’s nutritional requirements.

Given the inaccuracy of estimating energy requirements and negative consequences of both over and underfeeding patients, PICU nutritional guidelines currently endorse precise and personalized determination of energy needs by indirect calorimetry (50). The ongoing physical growth and cognitive development of children suggest a need for early and adequate nutrition, supported by evidence that even short episodes of starvation during infancy result in permanent neurocognitive losses (29, 58, 59). One size does not fit most when it comes to delivery of calories and protein, and, in the absence of measured energy expenditure, patients may be easily misclassified as adequately fed when they are in fact over or underfed. This misclassification makes the relationship between energy adequacy and patient outcomes difficult to study. Indirect calorimetry is specifically recommended in the setting of outlier BMI (<5th percentile or >85th percentile), a >10% change in weight, prolonged ventilation, prolonged muscle relaxation, thermal injuries, oncologic diagnosis, or neurologic injury with dysautonomia (50). Indirect calorimetry is labor intensive, cannot be completed in all patients, and is poorly reimbursed but can be targeted to these high-risk populations, where precise nutritional support is likely to alter patient outcome (50). In the absence of measured resting energy expenditure, clinicians rely on predictive equations to assess calorie requirements and biomarkers to monitor for return of growth. Persistently elevated CRP and decreased prealbumin may indicate ongoing catabolism, while normalization may reflect transition to an anabolic state with a concurrent increase in energy requirements (60, 61).

Current recommendations are that energy should be prescribed as a balanced mixture of carbohydrates and lipids after appropriate provision of protein to preserve lean muscle mass (1). As previously discussed, the delivery of additional carbohydrates does not inhibit stress response gluconeogenesis and may result in hyperglycemia and its morbidities (poor wound healing, immune dysfunction, prolonged mechanical ventilation, and damage to the endothelial glycocalyx) (62–66). Lipid metabolism and turnover are increased in critical illness as fatty acids are used as a primary fuel source (64). Excessive carbohydrates are converted to lipids but generate carbon dioxide in the process, which may prolong mechanical ventilation (60). Infants and children generally have limited fat stores and are susceptible to the development of essential fatty acid deficiencies as early as 1 week into critical illness, if not receiving sufficient lipids. Lipids are generally limited to 30–40% of total calories. Balanced nutritional strategies provide sufficient macronutrients, while avoiding acute complications of excess protein, carbohydrate, and lipids.

## Adequate Enteral Nutrition is Associated with Reduced Mortality in pARDS

### Evidence for Early Nutritional Support

Prioritization of early EN is associated with improved tolerance of future EN and a reduction in morbidity from sepsis (3, 36). Murine studies demonstrated improved preservation of intestinal histoarchitecture and barrier function and improved local and remote organ immune function, with concurrent reduction in bacterial translocation and endotoxemia with the use of early EN (19, 67–70). Clinical research has demonstrated decreased mortality when EN is started within 48 h (30, 36). Specifically, one large study of >5000 patients with a PICU stay >96 h demonstrated a decrease in mortality associated with provision of at least 25% of goal calories within 48 h (Table 1) (36). Adult studies have examined starting EN within 6 h of ICU admission without complication (71). These lines of evidence support the preferential use of EN in pARDS when it is safe to do so, a recommendation supported by the 2015 Pediatric Acute Lung Injury Consensus Conference (72).

### Evidence for Dose of Enteral Nutrition

Multiple retrospective studies demonstrate mortality benefit with improved adequacy of energy and protein provided during pediatric critical illness (Table 1) (2, 3, 36). In a prospective, international study of nutritional practices in 500 children ventilated for >48 h, Mehta et al. found that patients who received less than ⅓ of prescribed energy on average during the first 10 days after admission to the PICU had higher odds of mortality (3). An increase in prescribed energy by one tertile significantly decreased the odds of mortality. This relationship was only observed in patients with increased energy fed by the enteral route. ICUs, where a feeding guideline was in place, had overall lower hospital-acquired infection rates (3). Wong et al. examined 107 children with pARDS and identified that adequate energy and protein intake were associated with reduced mortality, while protein adequacy was also associated with increase ventilator-free days (4). These lines of evidence suggest that optimizing safe EN, rather than delivery of energy or protein *via* other routes of nutrition, is important for improving outcomes of pARDS.

Despite data regarding early nutritional adequacy and improved mortality, patients frequently fail to meet their calorie goals during the first week of illness with median intake generally falling at 40–75% due to multiple barriers to provision of EN (3, 5–10). Mechanisms for poor patient outcome in the setting of under nutrition relate to both nutritive and non-nutritive sequelae of inadequate EN. Direct nutritive consequences to under nutrition are largely due to inadequate protein substrate for production of acute phase and immune proteins, with subsequent loss of lean body mass. This may lead to loss of both respiratory and cardiac muscle and difficulty with ventilator weaning (Figure 1). Non-nutritive benefits of EN include improved intestinal barrier function, improved immune function, and maintenance of intestinal microbiome diversity. Appropriate targets to monitor these non-nutritive benefits are under development. Therefore, “optimal” nutrition can be defined in many ways depending on the target to define efficacy: sufficient energy and protein to meet metabolic demand, sufficient energy and protein to maintain lean body mass and functional recovery, sufficient energy and protein to maintain neurocognitive development, sufficient EN to maintain intestinal barrier functions, and adequate enteral composition to maintain microbiome diversity. The appropriate nutritional target in children with ARDS is not defined.

## Overfeeding and Underfeeding are Associated with Worse Outcome in Pediatric Critical Illness

Studies in adults and animals suggest negative impact of excessive nutrition with difficulty with ventilator weaning and either a mortality benefit or non-inferiority with restricted caloric intake (Table 2) (73–79). Several retrospective studies in critically ill children identify overfeeding occurring in PICU patients with negative clinical consequences (53, 80). Overfeeding is associated with delayed ventilator weaning, lipogenesis, hepatic dysfunction, hyperglycemia, increased mortality, and prolonged hospitalization. Complications associated with underfeeding included delayed ventilator weaning, impaired protein synthesis, organ failure, and an increased risk of sepsis (7, 10, 24, 32). Several potential mechanisms exist for the negative impact of overfeeding on patient outcome, such as increase in carbon dioxide production, increased intolerance of EN and parenteral nutrition (PN), refeeding syndrome, azotemia and metabolic acidosis from excess protein administration, hepatic steatosis from excess glucose delivery, hyperglycemia, hypertriglyceridemia, and on the cellular level the suppression of autophagy (53, 62, 81, 82). Administration of PN early in the course of critical illness may suppress autophagy, a necessary form of programmed cell death, which aids in removal of damaged proteins and mitochondria and is thought to play a role in recovery after organ failure (83).

**Table 2:** **Key randomized, controlled trials in critical care nutrition 2006–2016**.

Study	Patient population	Intervention/comparison	Outcomes
**Dose, route, and timing**			
Parenteral nutrition			
Fivez et al. (84)	Multicenter RCT involving 1440 critically ill children	PN at 24 h versus 1 week (EN initiated in both groups)	No difference in mortality. Fewer infections in late PN (10.7 versus 18.5%) group, shorter duration of mechanical ventilation (*p* = 0.001), fewer patients required renal replacement therapy (*p* = 0.04), shorter ICU stays (*p* = 0.001)
Harvey et al. (85)	Multicenter RCT of 2400 critically ill adults	Early PN versus Early EN up to 5 days after ICU admission in adults who could be enterally fed	No difference in 30- (33.1 versus 34.2%, *p* = 0.57) or 90-day mortality (*p* = 0.4). Reduced hypoglycemia, vomiting in the early PN group. No difference in treated infectious complications
Doig et al. (86)	Multicenter RCT of 1372 critically ill adults	Early PN versus standard care for patients with relative contraindications to early EN	No difference in 60-day mortality (21.5 versus 22.8%, *p* = 0.6). Early PN patients required fewer days of mechanical ventilation and experienced less muscle wasting
Heidegger et al. (87)	Multicenter RCT of 153 critically ill adults meeting <60% of caloric needs by EN on ICU day 3	Supplemental PN versus EN alone on days 4–8	Supplemental PN associated with higher percentage of energy target and fewer nosocomial infections
Casaer et al. (88)	Multicenter RCT of 2328 critically ill adults	PN at 48 h versus 1 week (EN initiated in both groups)	No difference in mortality. Fewer ICU infections (22.8 versus 26.2%, *p* = 0.008) in late PN group, smaller proportion of patients with >2 days of mechanical ventilation, median reduction of 3 days for renal replacement therapy (*p* = 0.008)
Dose of macronutrients			
Arabi et al. (89)	Multicenter RCT of 894 critically ill adults	Permissive underfeeding of non-protein calories (40 to 60% of goal) versus standard enteral feeding (75–100% of goal)	No difference in 90-day mortality (27.2 versus 28.9%, RR 0.94). No significant between-group differences for feeding intolerance, diarrhea, ICU-acquired infections, or length of stay
Braunschweig et al. (90)	Single-center RCT of 78 adults with acute lung injury	Intensive medical nutrition (>75% goal) versus standard nutritional support (~55% goal)	Significantly greater hospital mortality in intensive group (40 versus 16%, *p* = 0.02)
Rice et al. (91)	Multicenter RCT of 1000 adults with acute lung injury	Early trophic versus full enteral feeding	No difference in 28-day ventilator-free days (14.9 versus 15.0, *p* = 0.89) or 60-day mortality (23.2 versus 22.2%, *p* = 0.77). No differences in infectious complications between the groups. Initial trophic feeds were associated with less feeding intolerance
Gastric versus post-pyloric feeds			
Davies et al. (92)	Multicenter RCT of 181 critically ill and intubated adults with elevated gastric residuals	Continuation of gastric feeds versus transition to post-pyloric feeds	No clinically significant difference
Acosta-Escribano et al. (93)	Single-center RCT of 104 adults with severe traumatic brain injury	Gastric versus post-pyloric feeds	Lower incidence of pneumonia in post-pyloric groups OR 0.3 (95% CI 0.1–0.7, *p* = 0.01) and higher percentage of nutrional needs met (92 versus 84%, *p* < 0.01)
Hsu et al. (94)	Single-center RCT of 121 critically ill adults	Gastric versus post-pyloric feeds	Post-pyloric feeds associated with earlier achievement of nutritional goals, less vomiting, and less pneumonia
Early enteral nutrition			
Khorasani and Mansouri (95)	Single-center RCT of 688 burned children	EN at 3–6 h versus at 48 h	Early EN associated with decreased length of stay and mortality (12 versus 8.5%, *p* < 0.05)
Continuous versus bolus enteral nutrition			
MacLeod et al. (96)	Single-center prospective RCT of 164 critically ill adult trauma patients	EN as q 4 h boluses versus continuous drip	Intermittent regimen reached goal quicker with no difference in complications
**Glycemic control**			
NICE-SUGAR study investigators et al. (97)	Multicenter RCT of 6104 critically ill adults	Intensive glucose control (81–108 mg/dL) versus conventional contol (<180 mg/dL)	Higher mortality (OR 1.14; 95% CI, 1.02–1.28; *p* = 0.02) and more hypoglycemia (6.8 versus 0.5%, *p* < 0.001) in the intensive group
Vlasselaers 2009 (98)	Single-center RCT of 317 critically ill infant and 383 critically ill children (700 total)	Intensive normoglycemia with target glucose 50–79 (infant)/70–100 (children) versus target glucose <214 mg/dL	Lower mortality (3 versus 6%, *p* = 0.013) despite more hypoglycemia (25 versus 1%, *p* = 0.001) in the intensive group
Van den Berghe et al. (99)	Single-center RCT of 1200 critically ill adults (medical)	Intensive glucose control (80–110 g/dL) versus conventional control (<180 mg/dL)	No difference in mortality but decreased acute kidney injury (5.9 versus 8.9%, *p* = 0.04), earlier weaning from mechanical ventilation (*p* = 0.03), and shorter hospital stay in intensive group (*p* = 0.05)
**Immune modulation**			
Glutamine and selenium, antioxidants			
Ziegler et al. (100)	Multicenter RCT of 150 critically ill adults	Alanyl–glutamine dipeptide (0.5 g/kg/d), proportionally replacing amino acids in PN versus standard PN (EN initated as tolerated)	No difference in clinical outcomes
Pérez-Bárcena et al. (101)	Multicenter RCT of 142 critically ill adults (trauma)	l-alanyl–l-glutamine dipeptide (0.5 g/kg body weight/day) supplementation for 5 days versus standard PN	No difference in clinical outcomes
Heyland et al. (102)	Multicenter RCT of 1223 critically ill adults	(l-alanyl–l-glutamine dipeptide at 0.5 g/kg body weight/day PN supplementation and 42.5 g of alanyl–glutamine and glycine–glutamine dipeptides EN supplementation) versus (500 μg of selenium intravenously + 300 μg of selenium, 20 mg of zinc, 10 mg of beta carotene, 500 mg of vitamin E, and 1500 mg of vitamin C orally) versus combination versus placebo	Higher mortality in those receiving glutamine (OR 1.28, CI 1.00–1.64, *p* = 0.05)
Carcillo et al. (103)	Multicenter RCT of 293 critically ill children	Enteral zinc, selenium, glutamine and IV metoclopramide (ZSGM) versus enteral WHEY protein and IV saline up to 28 days of ICU stay	No differences in time until first episode of nosocomial infection/sepsis (median WHEY 13.2 days versus ZSGM 12.1 days, *p* = 0.29) or the rate of nosocomial infection/sepsis (4.83/100 days WHEY versus 4.99/100 days ZSGM, *p* = 0.81)
Andrews et al. (104)	Multicenter RCT of 502 critically ill adults	Parenteral glutamine (20.2 g/day) or selenium (500 μg/day) or both for up to 7 days versus placebo	No affect on new infections or mortality except for a reduction in infections for patients receiving selenium for 5 or more days (OR 0.53, CI 0.30–0.93).
Angstwurm et al. (105)	Multicenter RCT of 189 adults with severe sepsis/SIRS	Selenium as 1000 μg of sodium-selenite over 30 min followed by continuous infusions of 1000 μg daily for 14 days versus placebo	Reduced mortality in the selenium group (OR 0.56; CI 0.32–1.00, *p* = 0.049)
Omega-3 fatty acids alone or in combination			
Grau-Carmona et al. (106)	Multicenter RCT of 159 critically ill adults	Total PN with a lipid emulsion containing 10% fish oil versus a fish oil-free lipid emulsion	Fish oil emulsion associated with decreased nosocomial infections (21.0 versus 37.2%, *p* = 0.035)
Kagan et al. (107)	Single-center RCT of 120 critically ill adults (trauma)	EN enriched with eicosapentaenoic acid, γ-linolenic acid, and antioxidants versus a non-enriched control formula initiated at time of admission	No significant difference in clinical outcomes
van Zanten et al. (108)	Multicenter RCT of 301 intubated adults	EN enriched with glutamine, omega-3 fatty acid, and antioxidants (experimental product, NV Nutricia, Zoetermeer) versus high-protein tube feed (Nutrison Advanced Protison, NV Nutricia, Zoetermeer)	No difference in infection rate and increased 6-month mortality associated with immunomodulatory EN (35 versus 54%, *p* = 0.04)
Jacobs et al. (22)	Multicenter RCT of 26 critically ill children with acute lung injury	EN supplemental with eicosapentaenoic acid, γ-linolenic acid, and antioxidants versus standard EN	Improved biochemical profile
Pontes-Arruda et al. (109)	Multicenter RCT of 115 critically ill adults	Immunomodulator EN with eicosapentaenoic acid and γ-linolenic acid (Oxepa) versus standard EN (Ensure Plus HN)	No significant difference in mortality but immunomodulatory EN associated with decreases in the severity of sepsis, cardiovascular failure, respiratory failure, mechanical ventilation, and length of stay
Radrizzani et al. (110)	Multicenter RCT of 326 critically ill adults	Immunomodulatory EN (Perative, 55% carbohydrate, 25% fat, 21% protein, 1.3 kcal/mL, containing per 100 mL: 0.8 g l-arginine, 0.15 g ω-3 fatty acids, 0.7 g ω-6 fatty acids, 2.9 mg vitamin E, 0.75 mg β-carotene, 2.2 mg zinc, and 7 μg selenium) versus PN (containing 59% carbohydrate, 23% fat, 18% protein, 1.2 kcal/mL)	No difference in mortality. Immunomodulatory EN associated with decreased progression to severe sepsis or septic shock (4.9 versus 13.1%, *p* = 0.022) and shorter ICU length of stay

## Adult ICU Guidelines

American Society of Parenteral and Enteral Nutrition (ASPEN) Guidelines of the Provision and Assessment of Nutrition Support Therapy in the Adult Critically Ill Patient recommend that patients with ALI/ARDS receive either trophic or full EN over the first week of hospitalization. Even in adults, there is uncertainty regarding timing and dose of nutrition therapy. Evidence exists that hypocaloric nutritional strategies, experienced by the majority of patients, may not benefit specific subsets of the critically ill, such as in the setting of mechanical ventilation, severe burns, and surgery (111–113). Patients at high nutritional risk, defined as either undernourished or overnourished at baseline may benefit from a more aggressive nutritional strategy (114). The intensive nutrition in acute lung injury (INTACT) study was designed to deliver >75% of goal energy and protein needs daily to adult ICU patients *via* EN from date of diagnosis of ARDS to hospital discharge as compared with standard nutrition support (Table 2) (90). The INTACT study found increased mortality (40 versus 16%, *p* = 0.02) with aggressive nutritional support (85% of estimated energy expenditure) when compared with standard therapy (55% of estimated energy expenditure) and was stopped early (90). While impossible to apply directly to pediatric patients due to their different metabolic needs and ongoing growth and neurocognitive development, these data reinforce the powerful role nutrition exerts on patient outcome and highlight the need for rigorous research to identify the optimal timing and dose of calories and protein, tailored to narrowly defined patient populations.

Individualized nutritional therapies that are “just right” and meet, but do not exceed, needs are currently recommended by ASPEN guidelines for nutritional prescription in critically ill children (50). “Optimal” energy and protein targets remain elusive, and response to nutrition delivery is likely related to baseline nutritional risk and current diagnosis. Increased utilization of non-invasive ventilation (NIV) in pARDS is associated with worse nutritional adequacy, and nutrition-related outcomes for patients managed with NIV are unknown (115). We do not know if potential benefits to NIV are outweighed by risks related to underfeeding. Underfeeding is harmful, but overfeeding is equally harmful. Multiple clinical trials are criticized for comparing very underfed to underfed patients, or for overfeeding patients. At the heart of these controversies lies the difficultly in determining in real-time calorie and protein needs and response to nutritional intervention. Advances in body composition assessments that are practical during pARDS in ultrasound, bioelectrical impedance, CT imaging, and functional outcomes assessments to monitor lean body mass close monitoring of lean body mass during pARDS a possibility, but require further study (116–119).

## Route of Nutrition

### Evidence for Gastric versus Post-pyloric Feeds

The decision to prescribe gastric or post-pyloric EN has limited evidence to provide guidance. A study of 74 critically ill children randomly assigned to gastric or post-pyloric feeds demonstrated no difference in complications, and patients receiving post-pyloric feeds received more of their prescribed calories (120). A large meta-analysis of adult patients with severe traumatic brain injury demonstrated a decreased risk of pneumonitis with post-pyloric feeds (121). Other researchers found an increased risk of gastrointestinal complications when post-pyloric feeds were used in septic patients or patients on epinephrine (Table 1) (37, 122). Institution-based policies regarding verification of enteral feeding tube position, particularly for post-pyloric feeds, may result in significant delays in initiation of feeds. No studies, so far, have specifically evaluated gastric versus post-pyloric feeds in the setting of pARDS.

### Evidence Regarding Parenteral Nutrition

Current standard practice in the US is to reserve PN for situations in which EN fails or is not possible. This practice is rooted in retrospective evidence linking PN to increased risks of sepsis and mortality when compared with EN alone (3). Unfortunately, the evidence to guide clinical decisions is limited by current practice patterns. Because PN is reserved for the sicker patient who cannot tolerate EN, retrospective studies are likely confounded by variables that predict both increased PN use and mortality. The Pediatric Early versus Late Parenteral Nutrition in Intensive Care Unit (PEPaNIC) study was an international, multicenter, randomized, and controlled trial comparing early versus late initiation of PN in critically ill children (Table 2) (123). The PEPaNIC Trial tested the relationship between early and later PN supplementation in 1440 children from newborn to 17 years of age and found no difference in mortality between the two groups (84). This study was not limited to children with pARDS. A significantly increased rate of hospital-acquired infections (18.5 versus 10.7%) was present in patients in the early PN group as compared with the late PN group (84). However, characteristics of the study design limit the generalizability of this study to pARDS patients. The macronutrient dose was not controlled in the study, and central line utilization was not reported in experimental and control groups. The study patients were heterogeneous, and not exclusive to pARDS. Questions remain whether early PN is of benefit in pARDS. The potential benefit and/or harm from early PN is unclear, and the current clinical focus is to provide sufficient energy and protein preferentially by the enteral route until further studies are completed.

Research in neonatal intensive care unit supports the early use of at least low rates of EN to support bowel health in combination with PN to meet metabolic needs with the added advantage of fewer interruptions in nutrient delivery when PN is used (124). The use of this combination therapy, beginning with the first hours to days of life in preterm infants, has been associated with improved growth, improved neurodevelopment, improved EN tolerance, and decreased morbidity at both intermediate (18 months) and long-term (5 year) follow-up (30, 58, 59, 125). It remains unclear if older or term infants with ARDS might benefit from a similar early PN strategy with regard to short-term clinical and long-term neurocognitive outcomes. Research in the adult patient population have demonstrated mixed results with regard to days of mechanical ventilation and morbidity, such as infection and renal insufficiency (78, 86, 88). This is consistent with previous literature suggesting improved outcomes with calorie restriction in the adult population (78). Given the ongoing growth and development of pediatric patients, it is reasonable to question whether combination therapy with EN and PN in children with pARDS will see similar benefits to those demonstrated in the NICU population. Despite the results of the PEPaNIC study, equipoise remains in the pARDS population. The optimal macronutrient dose, timing, and formulation of EN and PN support has yet to be elucidated as increasing evidence demonstrates links between the immune system, homeostasis, and nutritional intake (121, 126–129).

### Barriers to Nutritional Support

Barriers to adequate EN can be broadly categorized as medical contraindication, prescriber discomfort, and frequent interruption (7, 9, 130, 131). Subjective rather than objective decision-making continues regarding decisions to initiate, advance, or maintain EN. Medical contraindications can include a need for volume restriction, hemodynamic instability, and ill-defined feeding intolerance. Studies in hemodynamically unstable adults requiring vasopressors demonstrated lower mortality with early EN (132). While large database studies do not demonstrate increase in adverse intestinal outcomes in children fed enterally while on vasoactive infusions, children with hemodynamic instability who do experience complications of EN have worse outcomes than children not fed by the enteral route (38, 122). Provider concerns regarding complications of EN in the setting of hemodynamic instability are warranted (122). Research is needed to further clarify the risks of EN while on vasoactive infusions, to identify optimal dosing strategies in the setting of hemodynamic instability, and to develop biomarkers to monitor safety of EN so that we might minimize risk, minimize unnecessary practice variation, and maximizing EN to patients at low risk of intestinal complications.

Feeding intolerance is the most frequent cause of interruption to EN, particularly in the current practice environment of deferring PN in marked preference for full EN. There is immense variability in clinician assessment of feeding intolerance and frequently used clinical criteria, such as bowel sounds, abdominal exam, gastric residual volumes, and lactate levels have not been validated (9, 130, 133, 134). Feeding intolerance occurs in 43–57% of critically ill children (9, 135). Even the definition of feeding intolerance is widely variable, and the natural history of bowel function in critical illness is yet to be fully elucidated (136, 137). Delayed gastric emptying and poor intestinal motility are causes of feeding intolerance, and, if left untreated, are impediments to achievement of goal EN. Delayed gastric emptying occurs in up to 50% percent of critically ill children, yet remains under recognized as a source of feeding intolerance (138). Promotility agents are commonly used for both gastric and intestinal dysmotility during pediatric critical illness, but only erythromycin and metoclopramide are currently approved in the US. Newer promotility agents, such as cholecystokinin receptor antagonists, ghrelin, and methylnaltrexone, in the setting of opioid-induced dysmotility, require further research in children with pARDS, but show promise to improve EN tolerance (138, 139). The development of validated tools for the diagnosis and monitoring of feeding intolerance represents an opportunity to significantly improve care in PICU patients by decreasing barriers to nutritional support. Biomarker-based guidelines for the initiation, advancement, and maintenance of EN are under development (140). Depending on hospital policies and local practice patterns, procedures are also a frequent cause of held EN and interruption of nutritional support despite an absence of data to guide these decisions (9). NIV and intensive therapies, such as extracorporeal membrane oxygenation, are in particular associated with nutritional interruption (141, 142). In adult ICU’s, improved nutritional adequacy is reported when volume-based daily feeds are ordered, rather than an hourly rate (143). Volume-based orders accommodate 4–6 h of NPO status daily with the volume of EN delivered over 18–20 h, but larger studies are needed to understand the clinical outcome of a volume-based EN strategy in pARDS. A currently enrolling clinical trial, continuous versus bolus nasogastric feeding for mechanically ventilated pediatric patients (Clinical Trials ID: NCT02566070), will evaluate feeding intolerance and nutritional adequacy with two gastric feeding strategies. Consideration should be given to early initiation of a bowel regimen to prevent constipation and subsequent feeding intolerance while avoiding diarrhea and malabsorption. Implementation of an early EN guideline improved percent of goal energy and protein achieved in multiple retrospective studies, likely due to perceived emphasis on nutrition in a particular PICU (144, 145).

### Glycemic Control

Literature in the adult population demonstrated compelling early evidence for significant decreases in morbidity and mortality with tight glycemic control (146). Subsequent research has demonstrated no benefit, but these results are drawn into question by large differences in mean glucose level and the use of point-of-care glucose testing rather than the arterial blood gas analysis used in the initial study (147–149). The negative impact of poor glycemic control is supported by current pediatric research, but the ideal target for blood glucose ranges has yet to be established (98, 150). The Heart And Lung Failure – Pediatric INsulin Titration (HALF-PINT) trial (Clinical Trials ID: NCT01565941) is a multicenter, randomized clinical treatment trial comparing effectiveness of tight glycemic control to a target range of 80–110 mg/dL versus a target range of 150–180 mg/dL.

## The Gut as the Motor for ARDS

There are many research opportunities to improve nutritional supplementation for critically ill patients in general as well as specifically within the framework of pARDS. Emerging research continues to demonstrate the role of gut dysfunction in the development of ARDS (13, 151). Deitch proposed the “gut lymph” hypothesis, whereby, the injured gut allows translocation of bacteria and bacterial products and liberates cytokines and chemokines, which act *via* the lymphatic duct to activate alveolar macrophages and contribute to pathogenesis of ARDS (152). Clark and Coopersmith proposed the “intestinal crosstalk” theory whereby the intestinal microbiota, immune system, and intestinal epithelial barrier interact and, when dysregulated, worsen systemic inflammation (153–155). Loss of balance in this “crosstalk” may lead to ARDS and is supported by emerging animal and human data examining the microbiome in critical illness. Modulation of intestinal barrier function and microbiome remain novel targets to improve outcomes in pARDS.

**Figure 2:**
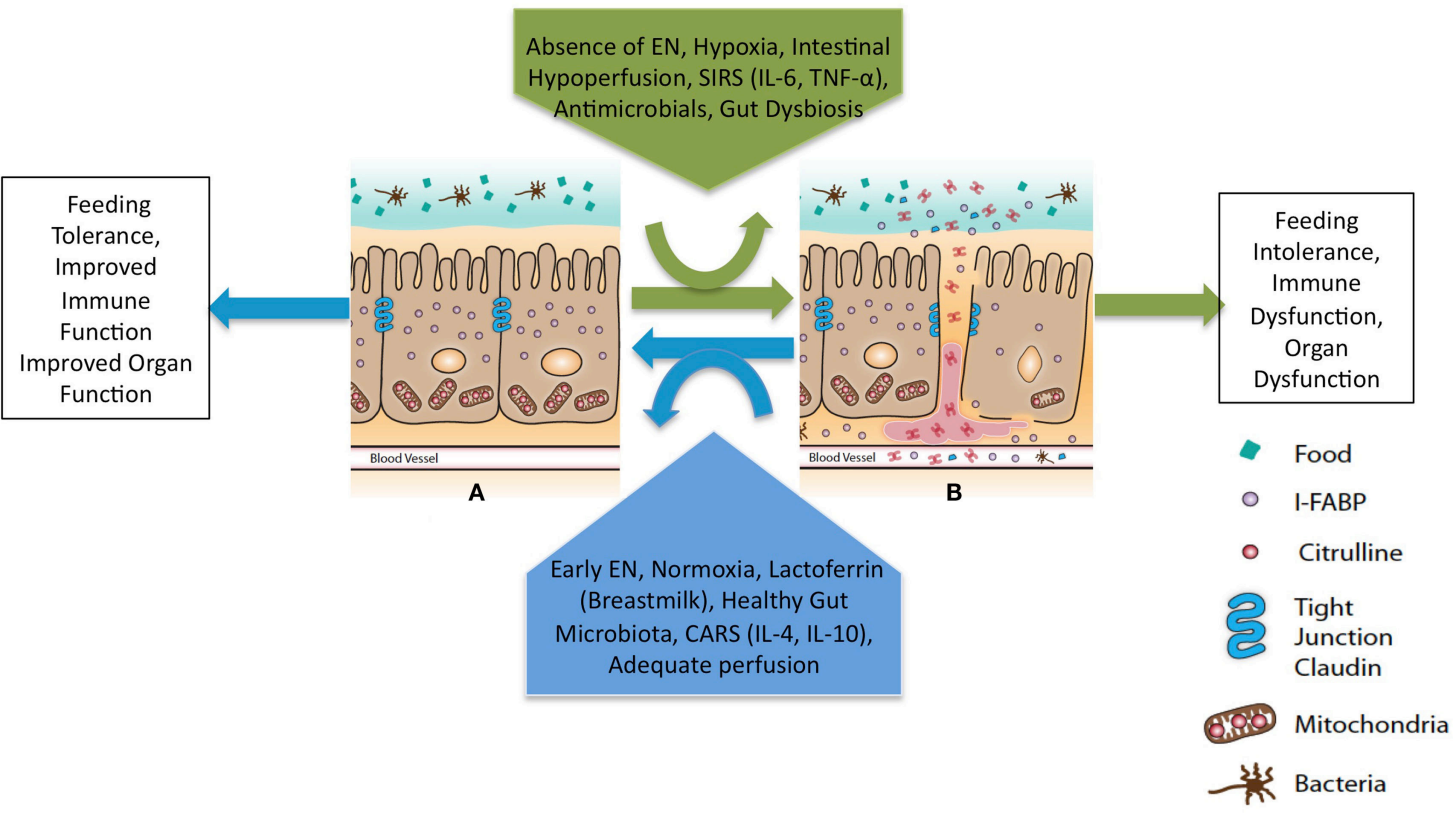
**Theoretical framework to maintain intestinal barrier dysfunction in pARDS**. EN, enteral nutrition; SIRS, systemic inflammatory response syndrome; CARS, compensatory anti-inflammatory response syndrome. Primary determinants of intestinal barrier function are the apical tight junction complex and the intestinal epithelial cells. Modifiable clinical factors known to modulate intestinal barrier function include EN and antimicrobials. Specific targets for perfusion and oxygenation to improve intestinal barrier function in the setting of pARDS are unknown. The balance of forces will lead to intestinal barrier **(A)** function or **(B)** dysfunction and a downstream clinical phenotype with either improved or worsened remote organ (lung) function.

Several minimally invasive plasma biomarkers show promise in guiding initiation, titration, and continuation of EN by objectively assessing intestinal epithelial structure and function as well as assessing for the translocation of bacteria and bacterial products (Table 3) (156–158). The intestinal epithelial barrier is a single-cell monolayer tasked with competing roles to absorb nutrients, interact with commensal organisms, and limit entrance of pathogens and their products (Figure 2) (159, 160). It is the largest surface in the body in contact with the external environment. Intestinal barrier dysfunction is associated with the pathogenesis of multiple organ dysfunction, translocation of bacteria and endotoxin, and loss of remote organ immune function (19, 159, 161). Enteral feeding of at least 15% of goal calories restores intestinal barrier function in murine models of critical illness (69). The minimum dose of EN required to maintain intestinal barrier function in children with pARDS is unknown. Biomarkers to monitor intestinal barrier function could guide provider decisions regarding the minimum necessary dose of enteral feeding. In a study of 20 children after cardiopulmonary bypass, biomarker concentrations were associated with symptoms of feeding intolerance (140). Biomarker-based decision rules for initiation, advancement, and maintenance of EN represent an opportunity to reduce practice variation and improve success in achieving and maintaining nutritional goals. The development of biomarkers to accurately and easily measure the catabolic and anabolic balance of the metabolic system is another area of ongoing research which will assist clinicians to more accurately develop nutritional prescriptions (46).

**Table 3:** **Minimally invasive plasma biomarker candidates to assess intestinal barrier function**.

Biomarker	Site specificity	Relevant data
Intestinal fatty acid-binding protein (I-FABP or FABP2)	Enterocytes of the small and large intestine	Plasma concentration correlates with histological phases of enterocyte injury after ischemia-reperfusion and is a marker of acute enterocyte damage (156, 157). Predictive of poor outcome if elevated after refeeding infants with NEC (158)
Liver fatty acid-binding protein (L-FABP)	Enterocytes of the small and large intestine, hepatocytes	Plasma concentration discriminates infants with sepsis versus NEC (162)
Claudin 3	Apical tight junction complex	Strong correlation between plasma claudin 3 and enterocyte tight junction loss in rat hemorrhagic shock model, human necrotizing enterocolitis (163, 164). Ubiquitous in lung tight junctions but lung injury does not cause elevation in plasma levels
Citrulline	Mature enterocytes of small intestine, colon	Manufactured in mitochondria of mature enterocytes. Validated as a biomarker for functional enterocyte mass in short bowel syndrome, HIV patients, stem cell transplant patients, graft-versus-host disease, and in children after bowel resection (165–171)
Trefoil Factor 3	Intestinal goblet and mucin cells	Differentiates between surgical and non-surgical NEC in preterm infants and assess disease activity in inflammatory bowel disease (162, 172)

*NEC, necrotizing enterocolitis; HIV, human immunodeficiency virus*.

## Intestinal Microbiome may Shape Immune Responses

Technological advances in computing power and analytic techniques over the last decade make the study of the human microbiome possible (173). In the human body, bacterial cells exceed human cells in number and perform many essential functions, such as production of short chain fatty acids and vitamins (vitamin K), which aid the human host (174). The developmental maturation of the human microbiome is poorly understood, but it reaches adult patterns by 2–4 years of age (175). Dysbiosis occurs when the symbiotic relationship between human and microbial populations is disturbed and may result in reduced microbiome diversity. Reduced microbiome diversity during infancy, a critical time period during microbiome development, may have lasting consequences on the development of several chronic diseases (176). Murine models where antibiotics are provided during infancy, demonstrate rapid and permanent alteration of metabolic phenotype *via* transient reduction in intestinal microbiome diversity (176–178). In contrast, an increased microbiome diversity in human infants enhances maturation of the intestinal mucosal immune system, which can influence LPS responsiveness at 1 year of age in humans (179). Thus, normal metabolic and immune imprinting by essential “keystone” microbes, which can be altered by early antibiotic exposures, may have long-term consequences on immune response to critical illness and risk of chronic illness. This emerging field has the potential to shift our targets with regard to control of both acute and chronic inflammation from modulation of human cells to modulation of the bacterial within us. The gastrointestinal microbiota is implicated in control of inflammation in the lung (180). Diet is a potent determinant of intestinal microbiome diversity and, in murine models, trumps genetic background (181). Carmody et al., demonstrated, in a murine model, that rapid changes in diet resulted in rapid shifts in microbial composition (181). As diet is an essential determinant of microbiome diversity and alters lung inflammation, dietary manipulation of the gastrointestinal microbiome may be a new target for treatment of pARDS. We do not know how medical diets alter the intestinal microbiome during pARDS, nor do we understand the short or long-term consequences of nutritional, probiotic, or pre-biotic interventions.

## Immunonutrition

Immunonutrition is well tolerated and results in improved pro-inflammatory cytokine profiles, but is not associated with clear clinical outcome benefits (182). While small single-center studies of isolated pharmaconutrients showed promise, multiple combination nutritional and pharmacologic additives in critically ill adults aimed at modulating the inflammatory and immune response have not shown benefits (Table 2) (183, 184). Specific supplements that have been explored include several antioxidants, arginine, glutamine, metoclopramide, ω-3 fatty acids, zinc, and selenium (103, 185–190). The randomized comparative effectiveness pediatric critical illness stress-induced immune suppression (CRISIS) prevention trial examined the effect in critically ill children of daily enteral zinc, selenium, glutamine, and IV metoclopramide on incidence of nosocomial infections. No difference was seen between the two groups, and the trial was stopped early due to futility (103). Multiple combination nutritional and pharmacologic additives in critically ill adults aimed at modulating the inflammatory and immune response have been trialed, but have generally not shown any benefits (50, 191). The optimal formulation of lipids is an area of active research with continued focus on the role of ω-3 polyunsaturated fatty acids (PUFAs).

## Omega-3 Polyunsaturated Fatty Acids

**Figure 3:**
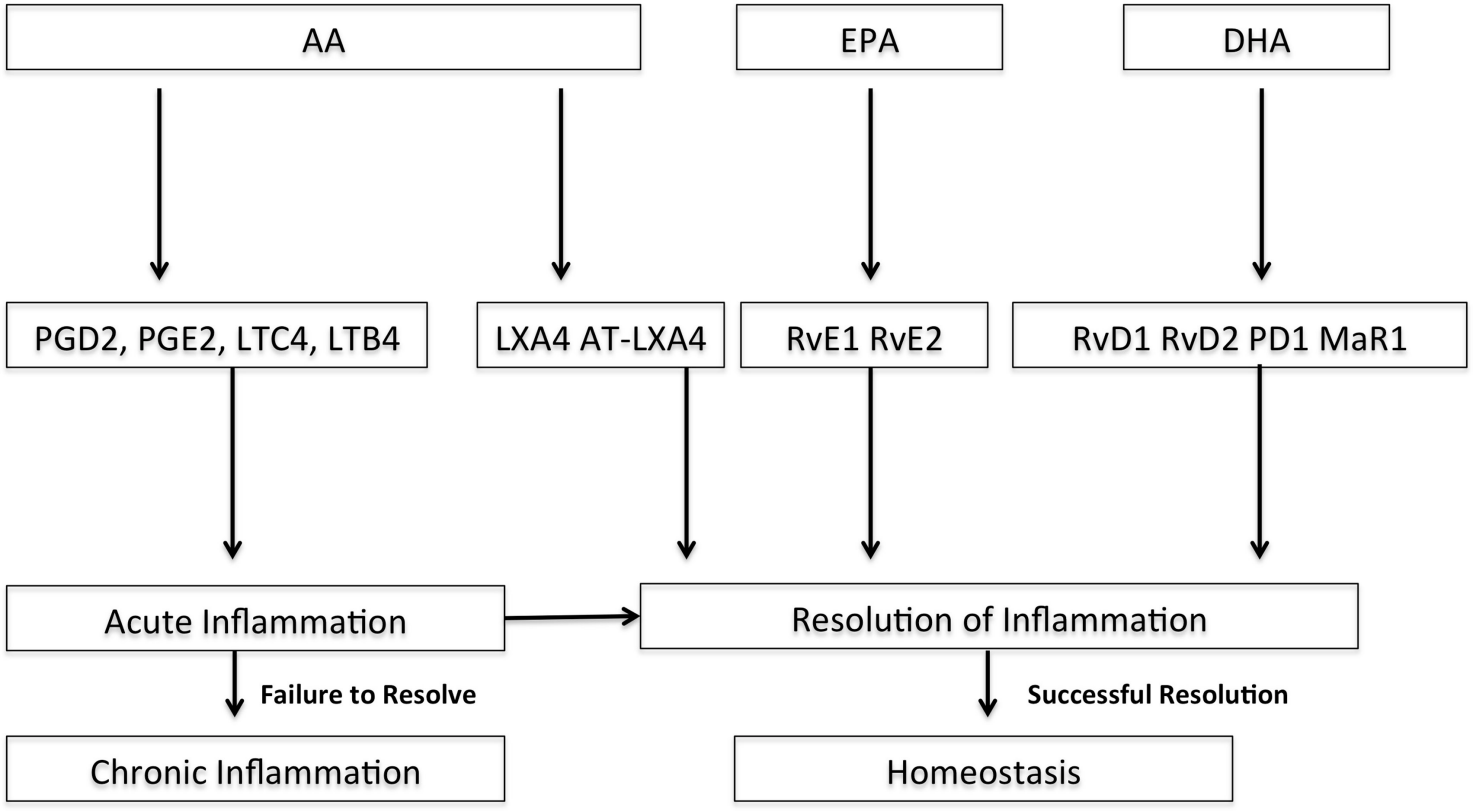
**Pathways of selected downstream lipid mediators derived from arachadonic acid (AA), docosahexaenoic acid (DHA), and eicosapentaenoic acid (EPA) for resolution of acute inflammation**. DHA and EPA are ω-3 fatty acids. Adapted with permission from Serhan and Petasis (198); PGD2, prostaglandin D2; PGE2, prostaglandin E2; LTC4, leukotriene C4; LTB4, leukotriene B4; LXA4, lipoxin A4; AT-LXA4, aspirin-triggered lipoxin A4; RvE1, resolvin E1; RvE2, resolvin E2; RvD1, resolvin D1, RvD2, resolvin D2; PD1, protectin D1; MaR1, maresin 1.

The use of ω-3 PUFAs as a nutritional adjuvant have evidence suggesting they may reduce the pro-inflammatory state in ARDS, but a recent systematic review and meta-analysis looking specifically at ARDS patients in the adult ICU found no benefit in clinical outcomes (107, 192–197). The rationale for ω-3 PUFAs is that they may be beneficial in ARDS as they would compete with ω-6 PUFAs, decrease the synthesis of pro-inflammatory eicosanoids, increase production of anti-inflammatory lipid mediators, such as resolvins and protectins, decrease in chemotaxis, decrease reactive oxygen species (ROS) and pro-inflammatory cytokines, and decrease leukocyte binding and activation through decreased expression of adhesion molecules (Figure 3) (192).

Jacobs et al. evaluated the feasibility of ω-3 PUFAs (eicosapentaenoic acid + γ-linolenic acid) in children with ALI and ARDS and examined the impact of ω-3 PUFAs on plasma phospholipid fatty acid concentrations (22). They found that delivery of nutrition enriched with eicosapentaenoic acid + γ-linolenic acid was feasible and resulted in an anti-inflammatory fatty acid profile (22). This study is supportive of future work in this area in children. Whether a change in anti-inflammatory fatty acid profile will improve clinical outcomes is unclear, but promising. Methodological challenges in several adult trials limit conclusions regarding ω-3 PUFAs in adult patients with ARDS (192). Specifically, studies are heterogeneous, and, in some, the control diet was pro-inflammatory.

Several ω-3 PUFA-derived mediators are potential targets to resolve the inflammatory response in the setting of pARDS (Figure 3). Alveolar edema and neutrophil recruitment and activation are early events in ARDS. Resolvins, protectins, and maresins are lipid-derived mediators, and several resolvins are emerging as potential therapeutic targets for resolution of ARDS (Figure 3) (20, 21). These resolvins are derived from the ω-3 PUFAs docosahexaenoic acid (DHA) and eicosapentaenoic acid (EPA). Eickmeier et al. found that aspirin-triggered resolvin D1 reduces mucosal inflammation and promotes resolution in a murine model of acute lung injury (21). Aspirin-triggered RV D1 decreased lung inflammation and promoted acute lung injury resolution shortly after injury in an hydrochloric acid model of ARDS by enhancing restitution of barrier integrity, decreasing circulating neutrophil–platelet heterotopic interactions, and regulating inflammatory mediators and nuclear factor-κB (NF-κB) activation (21). Seki et al. found that resolvin E1 protected mice from bacterial pneumonia and acute lung injury with pretreatment by decreased lung neutrophil accumulation, enhanced microbial clearance, decrease in lung pro-inflammatory mediators, and improved survival (20).

## Vitamin D

Recent evidence suggests an association between vitamin D levels and the risk of ARDS (199–201). Vitamin D deficiency is associated with impaired pulmonary function and increased incidence of viral and bacterial infections and inflammatory disease, including asthma and COPD (202–204). Mechanisms for these associations are unknown, but vitamin D plays a role in macrophage, lymphocyte, and epithelial cell function, critical to ARDS pathophysiology (200, 205).

## Summary

There are currently limited data to guide nutritional strategies in the critically ill pediatric patient and an absence of data targeted specifically at pARDS (Tables 2 and 3). Current strategies rely on the application of adult data, knowledge of the metabolic derangements caused by critical illness, and knowledge of the nutritional requirements of healthy children. The generally accepted strategy centers on the early identification of need for nutritional supplementation and early initiation of EN targeted toward fulfillment of nutritional goals and implemented by a collaborative, multidisciplinary treatment team. Indirect calorimetry is a recommended adjunct to ensure adequate, but not excessive, nutritional support is supplied. Early prescription of a bowel regimen to limit constipation while avoiding diarrhea is also recommended (50). PN is currently reserved for patients in whom EN is contraindicated or limited, though its routine use as a supplement to EN is an area ripe for research. Before we evaluate the role of immunonutrition to improve outcomes of pARDS, fundamental questions regarding protein and energy requirements during pARDS remain: if delivery should be based on premorbid nutritional risk and if under, normo, or even overfeeding might be beneficial at different stages of illness. Given the mortality benefit of nutritional adequacy on pARDS, it may prove challenging to identify benefits of adjuvant nutritional therapies, unless they are overlaid upon a foundation of adequate macronutrient delivery. Once fundamental questions regarding optimal timing, dose, and route of macronutrient delivery are answered for pARDS, investigations centered on immunonutrition to further improve patient outcomes are appropriate. Systematic investigations to determine the impact of each pharmaconutrient separately and in combination during pARDS are necessary.

In the future, intensivists will likely employ distinct and highly personalized nutritional therapies based on patient premorbid nutritional risk, admission diagnosis, severity of illness, measured energy expenditure, active monitoring of lean body mass, and the composition of patient intestinal microbiome. A select group of high-risk patients are likely to experience benefit from an intensive and personalized, titrated nutritional plan. Long-stay patients, patients with ARDS, sepsis, burns, or pre-existing severe malnutrition are most likely to have improved outcomes if nutrition is closely monitored. Intensive nutritional therapies will likely be titrated daily to meet energy and protein needs. Nutritional therapies, monitoring, and impact on meaningful patient outcomes are likely to change dramatically in the next decade as we understand the role of the microbiome in regulating both intestinal health and also the inflammatory response to pARDS. We may require nutritional or “metabolic” teams to precisely tailor metabolic support to meet, but not exceed, energy, protein, and micronutrient needs, preserve lean body mass, the microbiome, the virome, and intestinal barrier functions. Nutrition is fundamental to provision of critical care services, and, with an understanding of the complex role it plays in illness and health, we will be able to design effective clinical trials of nutritional interventions as primary therapies for pARDS.

## Author Contributions

Dr. BW and Dr. KT contributed equally to the conception, review of literature, writing, and editing of this manuscript. Both authors approve of the final submitted version of this manuscript.

## Conflict of Interest Statement

Dr. KT has received an investigator initiated grant from the Baxter Corporation in support of research to perform a clinical trial of early versus later PN in mechanically ventilated children. Funds provided by Baxter do not support her effort on the project, but provide project funds. BW declares no conflict of interest.
